# A case of IgG4-related anterior mediastinal sclerosing disease coexisting with autoimmune pancreatitis

**DOI:** 10.1186/s40792-020-00939-1

**Published:** 2020-07-23

**Authors:** Hiroshi Matsui, Takahiro Utsumi, Natsumi Maru, Yohei Taniguchi, Tomohito Saito, Haruaki Hino, Mitsuaki Ishida, Koji Tsuta, Tomohiro Murakawa

**Affiliations:** 1 Department of Thoracic Surgery, Kansai Medical University Hospital, Osaka, Japan; 2 Department of Pathology and Laboratory Medicine, Kansai Medical University Hospital, Osaka, Japan

**Keywords:** IgG4-related disease, Anterior mediastinal tumor, Steroid

## Abstract

**Background:**

IgG4-related disease (IgG4-RD) is a systemic fibro-inflammatory condition that predominantly involves exocrine organs. Concerning its thoracic presentation, it often manifests as interstitial lung disease or fibrosing mediastinitis. It is very rare for IgG4-RD to form a well-defined mass in the anterior mediastinum, mimicking an encapsulated thymoma.

**Case presentation:**

An 82-year-old man with autoimmune pancreatitis under treatment with oral corticosteroids was found to have peripancreatic lymphadenopathy on computed tomography. Subsequent positron emission tomography revealed abnormal uptake (maximal value 3.6) by a thymic mass as well as the peripancreatic lymph nodes. Exacerbation of IgG4-RD was suspected, and we increased the oral steroid dosage. As a result, the peripancreatic lymph nodes, and the anterior mediastinal mass, decreased in size. The thymic mass was suspected to be an encapsulated thymoma because of its lobulated shape, degree of fluorodeoxyglucose accumulation, and response to steroids, and the patient was referred to our department. The serum anti-acetylcholine receptor antibody test was negative. A thoracoscopic tumor resection was performed as diagnostic therapy. Histopathological analysis revealed dense lymphoplasmacytic infiltration with sclerotic stroma within the tumor. Immunohistochemical analysis revealed abundant IgG4-positive plasma cell infiltrates and over 50% IgG4/IgG-positive plasma cells. We did not see either keratin-positive thymocytes or terminal deoxynucleotidyl transferase-positive lymphocytes. Furthermore, deviation in the kappa chain and lambda chain-positive plasma cells was not noted. Accordingly, IgG4-related sclerosing disease was diagnosed.

**Conclusions:**

IgG4-related sclerosing masses in the anterior mediastinum are very rare, and the effect of tumor resection on prognosis remains unclear. IgG4-RD had potentially been categorized as Castleman’s disease.

## Background

IgG4-related disease (IgG4-RD) is a systemic disease characterized by an elevated serum IgG4 level and lymphoplasmacytic infiltration of multiple organs, such as the pancreas, salivary glands, and biliary tract [1]. In the respiratory system, it commonly presents as pulmonary nodules, lymphadenopathy, or sclerosing mediastinitis [2]. We present a case of IgG4-related disease forming a mass in the anterior mediastinum.

## Case presentation

**Fig. 1:**
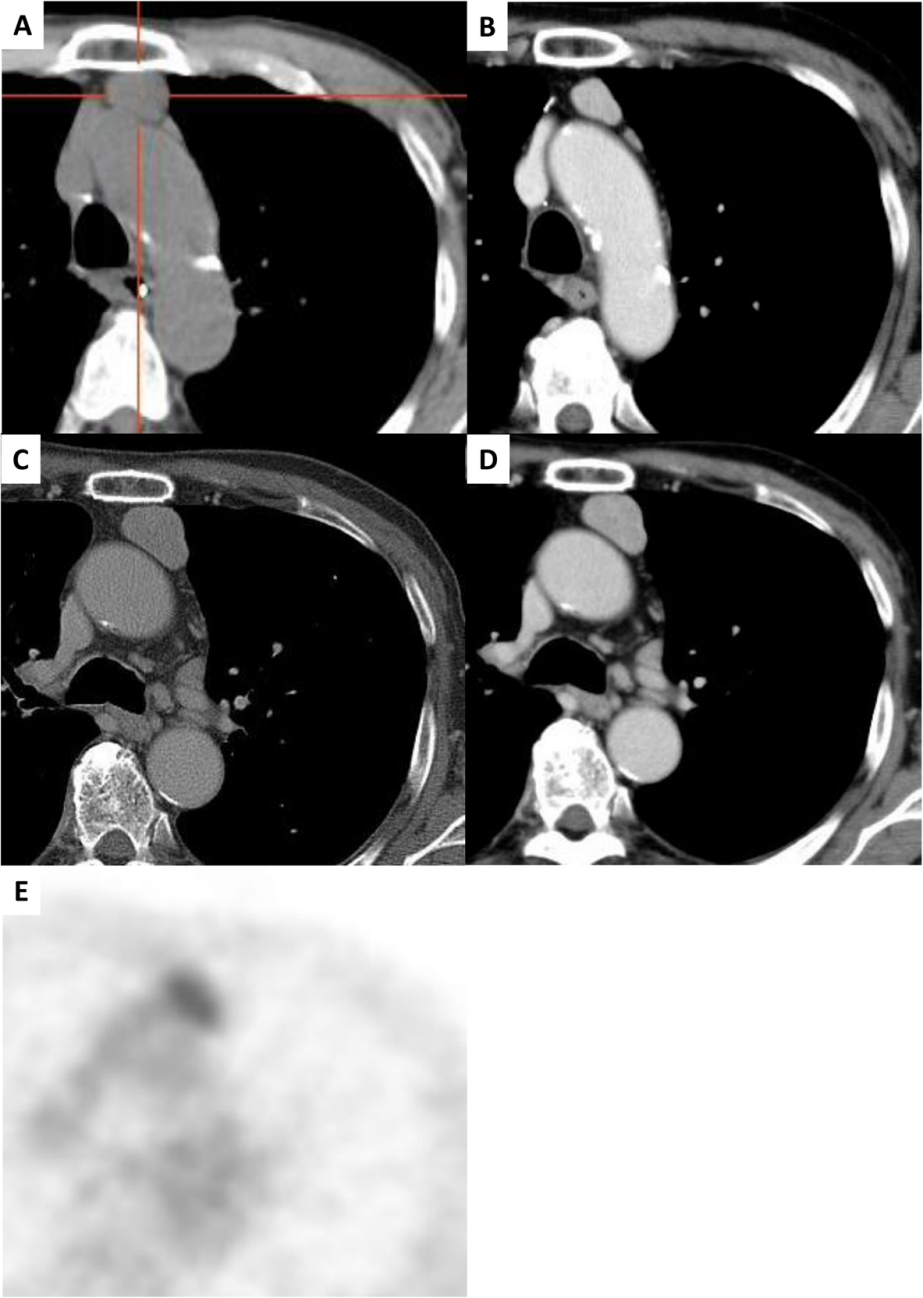
Anterior mediastinal mass. Chest computed tomography (CT) revealed a 2.5-cm well-defined homogenous mass in the anterior mediastinum at the time of diagnosis with autoimmune pancreatitis (**a**), and the tumor decreased in size to 2 cm on CT with oral steroid treatment 6 years ago (**b**). The tumor had regrown to 3 cm at the time of referral to our department on CT (**c**, **d**), and positron emission tomography showed a high maximum standardized uptake of 3.6 by the anterior mediastinal mass lesion (**e**)

An 82-year-old man was referred to our department with an anterior mediastinal mass. Eleven years earlier, the patient had been diagnosed with IgG4-related disease (IgG4-RD) from autoimmune pancreatitis and had been taking oral steroid treatment. At the initial diagnosis of IgG4-RD, a well-defined homogeneous anterior mediastinal mass was detected on chest computed tomography (CT); however, it temporarily decreased in size after starting oral steroid therapy (Fig. 1a, b). Serum level of IgE was normal (267.5 IU/mL), and he was negative for antinuclear antibody.

His medical history was notable for a history of asthma, multiple pulmonary nodules, and mediastinal lymphadenopathy. The latter was suspected to be Castleman’s disease 22 years ago. His serum interleukin-6 level was within the normal limits at the time (details unknown).

On this occasion, 5 months before surgery, peripancreatic lymphadenopathy without enlargement of pancreas was detected by CT, and subsequent positron emission tomography revealed abnormal uptake (maximal value 3.6) by the thymic mass as well as peripancreatic lymph nodes (Fig. 1c–e). Exacerbation of his IgG4-RD was suspected and a dose escalation of oral steroids from 2.5 to 10 mg/day was prescribed.

The anterior mediastinal mass was suspected to be an encapsulated thymoma because of its lobulated shape, degree of fluorodeoxyglucose accumulation, response to steroids, moderate enhancement on contrast CT, and the gradual enlargement of the tumor. The patient was therefore referred to our department.

He had no systemic symptoms, and his physical examination was normal. He had an elevated level of IgG4 at 715 mg/dL and soluble interleukin-2 receptor at 604 U/mL. The rest of his laboratory data were normal, including serum C-reactive protein and anti-acetylcholine receptor antibody. We performed tumor resection by video-assisted thoracic surgery. The tumor had not invaded the surrounding tissues. The operative findings were compatible with encapsulated thymoma, and the tumor was completely excised. His postoperative course was uneventful, and he was discharged on postoperative day 7. The levels of IgG4 and soluble interleukin-2 receptor after operation were 307 mg/dL and 403 U/mL, respectively.

**Fig. 2:**
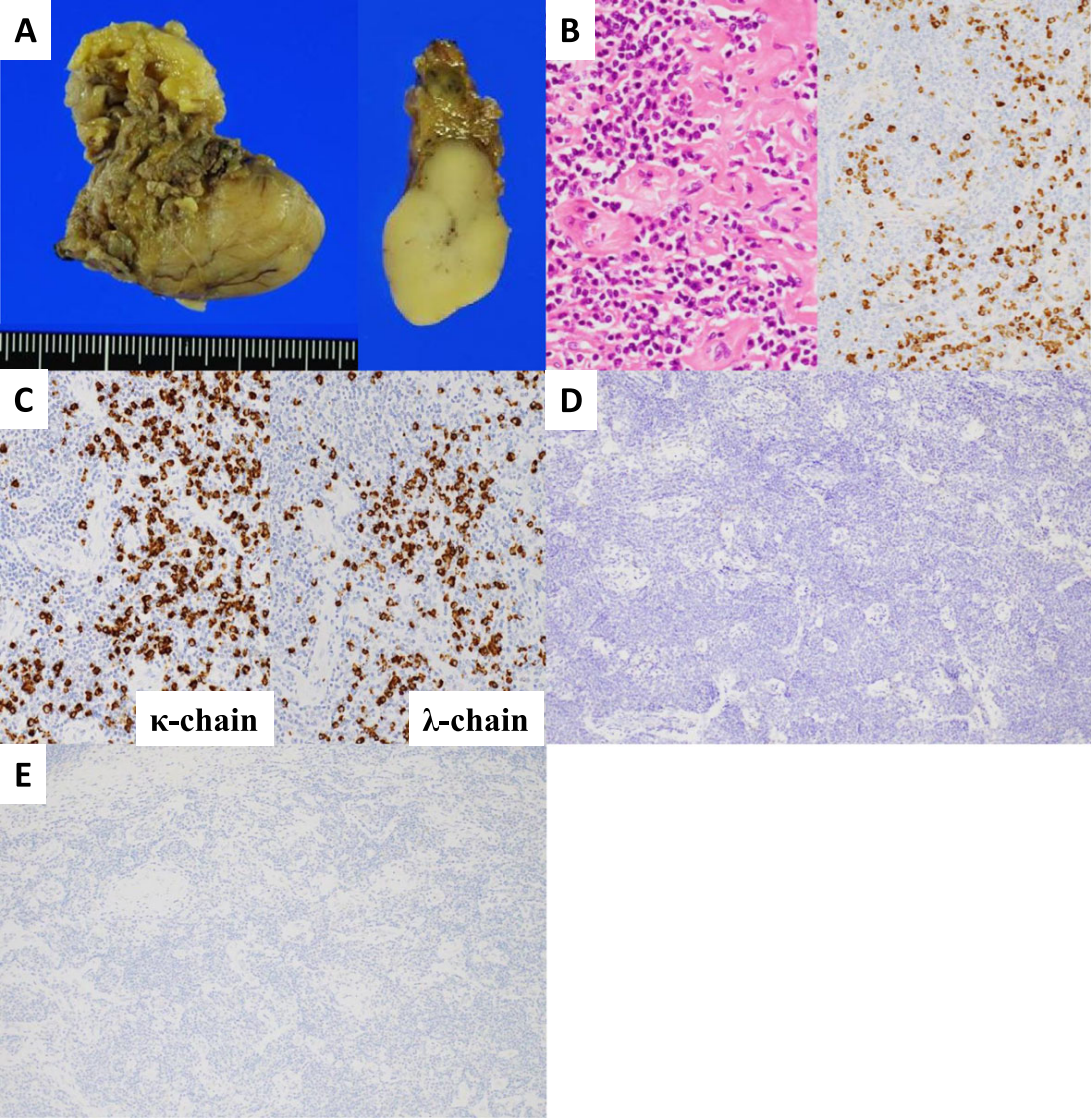
Pathological findings. A macroscopic view of the resected tumor, which is a lobulated, well-defined, white solid mass with one section (**a**). Histopathological findings of the specimen showed dense lymphoplasmacytic infiltration with sclerotic stroma (hematoxylin and eosin staining, 400×), and over 50% of IgG4/IgG-positive plasma cells (immunostaining, 200×) (**b**). No deviation in kappa chain- and lambda chain-positive plasma cells was noted (**c**, 200×), and neither keratin-positive thymocytes (**d**, 40×) nor terminal deoxynucleotidyl transferase-positive lymphocytes (**e**, 40×) were observed (immunohistochemical staining)

Macroscopically, the specimen was a lobulated, well-defined, white, solid mass (Fig. 2a). The final histopathological analysis revealed dense lymphoplasmacytic infiltration with sclerotic stroma within the tumor. Immunohistochemical examination revealed abundant IgG4-positive plasma cell infiltrates and more than 50% IgG4/IgG-positive plasma cells (Fig. 2b). No deviation in the kappa chain- and lambda chain-positive plasma cells was noted (Fig. 2c). Furthermore, neither keratin-positive thymocyte nor terminal deoxynucleotidyl transferase-positive lymphocytes were observed (Fig. 2d, e). Accordingly, IgG4-related sclerosing disease was diagnosed. Same dose of oral steroids was prescribed after surgery without tapering, and the condition of the IgG4-RD remains stable without exacerbation at 1 year follow-up.

## Discussion

IgG4-RD was first reported as a systemic disease in 2001 when patients with sclerosing pancreatitis were observed to have elevated serum IgG4 concentrations [3]. Since then, it has been recognized as an immune-mediated condition involving several organs, particularly exocrine organs such as the pancreas, salivary glands, and biliary tract [1]. Several studies have reported that intrathoracic involvement may be seen in about 10 to 50% [2, 4, 5], which commonly includes interstitial lung disease, inflammatory pseudotumors, fibrosing mediastinitis, and lymphadenopathy. The formation of a mass in the anterior mediastinum, mimicking an anterior mediastinal tumor, is very rare.

The differential diagnosis of an anterior mediastinal mass includes thymoma, germ cell tumor, and mucosa-associated lymphoid tissue (MALT) lymphoma [6]. It is not necessary to obtain the preoperative diagnosis of an anterior mediastinal tumor, given the risk of dissemination by a needle biopsy [7]. Germ cell tumors usually show heterogeneous images on chest CT, and steroid treatment is not effective. MALT lymphomas present homogeneous images and occasionally respond to steroids, but it is very rare. From the clinical findings, encapsulated thymoma was considered the most likely diagnosis.

IgG4-RD is diagnosed based on clinical and histopathological findings. Umehara H et al. proposed the following comprehensive diagnostic criteria for IgG4-RD: (1) organ involvement, (2) high serum IgG4 (> 135 mg/dl), (3) histological features such as IgG4-positive plasma cells > 10/high power field and IgG4-positive/IgG-positive cells > 40% in the affected lesion [8]. The present case meets the comprehensive diagnostic criteria for IgG4-related disease; however, Castleman’s disease sometimes satisfies these criteria [9, 10]. IgG4-RD also shares pathological characteristics with storiform fibrosis and obliterative phlebitis, so when there are few findings of storiform fibrosis or obliterative phlebitis as in this case, it is very difficult to differentiate between the two diseases only based on the pathological findings. Castleman’s disease is a polyclonal lymphoproliferative disorder, which is classified into two types: unicentric and multicentric [11]. Unicentric Castleman’s disease is characterized by localized disease confined to a single site. On the other hand, multicentric Castleman’s disease (MCD) involves lymphoid hyperplasia at multiple sites. MCD sometimes accompanied by the elevation of serum IgG4 levels and infiltration of IgG4-positive plasma cells in the affected organs. In MCD, interleukin-6 (IL-6) plays an important role, which is considered to be closely associated with its clinical presentation, with symptoms such as fever, weight loss, anemia, and elevated C-reactive protein (CRP) [12]. Differentiating between IgG4-RD and MCD by clinical characteristics alone is challenging. Sasaki T et al. reported that the pattern of organ involvement, atopic history, levels of IgA, and CRP were quite distinctive, and they suggested that the combination of involved orbits, lacrimal glands, salivary glands or pancreas, and an atopic history, IgA < = 330 mg/dL, and CRP < = 0.80 mg/dL may be useful for the differentiation of IgG4-RD from MCD [13]. Sato Y et al. reported that elevation of serum IgE was more frequently observed in lymph nodes of patients with IgG4-RD compared with those with MCD, and high levels of IL-6 and CRP may be important differential diagnostic markers for MCD apart from IgG4-RD [14]. In our case, the history of autoimmune pancreatitis and asthma, normal level of IgA (51 mg/dL at the first diagnosis of IgG4-RD) and CRP, high level of IgE (267.5 mg/dL at first diagnosis of IgG4-RD) and asymptomatic condition suggest IgG4-RD rather than MCD. His past history of multiple pulmonary nodules and mediastinal lymphadenopathy would have resulted from IgG4-RD and not Castleman’s disease.

Distinguishing IgG4-RD from MCD is important because their treatments are entirely different. IgG4-RD responds well to steroid therapy. Kamisawa et al. reported that the remission rate of patients with IgG4-related pancreatitis, receiving glucocorticoids was 98% [15]. On the other hand, MCD can be resistant to such treatment and is often treated with an anti-IL-6 agent [12]. In our case, the response to steroids might be another clue to the diagnosis of IgG4-RD.

## Conclusions

We described a case with an IgG4-related anterior mediastinal sclerosing mass. It is very rare for IgG4-RD to form a well-defined mass in the anterior mediastinum, and it is usually difficult to differentiate it from a malignant tumor before surgery. It is not clear why IgG4-RD formed the tumor-like swelling. There has been no study or review of the prognosis of intrathoracic lesions of IgG4-RD, and the impact of surgical resection is unknown.

IgG4-RD may mimic MCD clinicopathologically, and this case was possibly earlier misdiagnosed as MCD. Therefore, we should be careful when diagnosing patients with IgG4-RD; it should be based on the detailed clinicopathological findings in addition to meeting the diagnostic criteria of IgG4-RD.

## Data Availability

All datasets presented in the main paper are available whenever possible.

## Abbreviations

IgG4-RD: IgG4-related disease
CT: Computed tomography
MALT lymphoma: Mucosa-associated lymphoid tissue
IL-6: Interleukin-6
CRP: C-reactive protein
MCD: Multicentric Castleman’s disease

## References

[1] Stone JH, Zen Y, Deshpande V (2012). IgG4-related disease. N Engl J Med..

[2] Corcoran JP, Culver EL, Anstey RM, Talwar A, Manganis CD, Cargill TN (2017). Thoracic involvement in IgG4-related disease in a UK-based patient cohort. Respir Med..

[3] Hamano H, Kawa S, Horiuchi A, Unno H, Furuya N, Akamatsu T (2001). High serum IgG4 concentrations in patients with sclerosing pancreatitis. N Engl J Med..

[4] Fei Y, Shi J, Lin W, Chen Y, Feng R, Wu Q (2015). Intrathoracic involvements of immunoglobulin G4-related sclerosing disease. Medicine (Baltimore).

[5] Hirano K, Kawabe T, Komatsu Y, Matsubara S, Togawa O, Arizumi T (2006). High-rate pulmonary involvement in autoimmune pancreatitis. Intern Med J..

[6] Carter BW, Okumura M, Detterbeck FC, Marom EM (2014). Approaching the patient with an anterior mediastinal mass: a guide for radiologists. J Thorac Oncol..

[7] Wilkins EW, Grillo HC, Scannell JG, Moncure AC, Mathisen DJ (1991). J. Maxwell Chamberlain Memorial Paper. Role of staging in prognosis and management of thymoma. Ann Thorac Surg..

[8] Umehara H, Okazaki K, Nakamura T, Satoh-Nakamura T, Nakajima A, Kawano M (2017). Current approach to the diagnosis of IgG4-related disease - Combination of comprehensive diagnostic and organ-specific criteria. Mod Rheumatol..

[9] Nakamura M, Iwamoto O, Chino T, Todoroki K, Kusukawa J (2016). Diagnostic dilemma of IgG4-related primary localized cervical lymphadenopathy associated with aberrant IL-6 expression level. Diagn Pathol..

[10] Zoshima T, Yamada K, Hara S, Mizushima I, Yamagishi M, Harada K (2016). Multicentric Castleman disease with tubulointerstitial nephritis mimicking IgG4-related disease: Two case reports. Am J Surg Pathol..

[11] Castleman B, Iverson L, Menendez VP (1956). Localized mediastinal lymphnode hyperplasia resembling thymoma. Cancer..

[12] Nishimoto N, Kanakura Y, Aozasa K, Johkoh T, Nakamura M, Nakano S (2005). Humanized anti-interleukin-6 receptor antibody treatment of multicentric Castleman disease. Blood..

[13] Sasaki T, Akiyama M, Kaneko Y, Mori T, Yasuoka H, Suzuki K (2017). Distinct features distinguishing IgG4-related disease from multicentric Castleman’s disease. RMD Open..

[14] Sato Y, Kojima M, Takata K, Morito T, Asaoku H, Takeuchi T (2009). Systemic IgG4-related lymphadenopathy: A clinical and pathologic comparison to multicentric Castleman's disease. Mod Pathol..

[15] Kamisawa T, Shimosegawa T, Okazaki K, Nishino T, Watanabe H, Kanno A (2009). Standard steroid treatment for autoimmune pancreatitis. Gut..

